# Expression and Roles of Individual HIF Prolyl 4-Hydroxylase Isoenzymes in the Regulation of the Hypoxia Response Pathway along the Murine Gastrointestinal Epithelium

**DOI:** 10.3390/ijms22084038

**Published:** 2021-04-14

**Authors:** Franziska Dengler, Sofia Sova, Antti M. Salo, Joni M. Mäki, Peppi Koivunen, Johanna Myllyharju

**Affiliations:** 1 Unit of Physiology, Pathophysiology and Experimental Endocrinology, University of Veterinary Medicine, 1210 Vienna, Austria; 2 Faculty of Biochemistry and Molecular Medicine, Biocenter Oulu, University of Oulu, 90220 Oulu, Finland; sofia.sova@oulu.fi (S.S.); antti.salo@oulu.fi (A.M.S.); joni.maki@oulu.fi (J.M.M.); peppi.karppinen@oulu.fi (P.K.); johanna.myllyharju@oulu.fi (J.M.); 3 Institute of Veterinary Physiology, University of Leipzig, 04103 Leipzig, Germany

**Keywords:** jejunum, colon, caecum, epithelium, ddPCR, HIF, HIF-prolyl 4-hydroxylase, mRNA

## Abstract

The HIF prolyl 4-hydroxylases (HIF-P4H) control hypoxia-inducible factor (HIF), a powerful mechanism regulating cellular adaptation to decreased oxygenation. The gastrointestinal epithelium subsists in “physiological hypoxia” and should therefore have an especially well-designed control over this adaptation. Thus, we assessed the absolute mRNA expression levels of the HIF pathway components, *Hif1a*, *HIF2a*, *Hif-p4h-1*, *2* and *3* and factor inhibiting HIF (*Fih1*) in murine jejunum, caecum and colon epithelium using droplet digital PCR. We found a higher expression of all these genes towards the distal end of the gastrointestinal tract. We detected mRNA for *Hif-p4h-1*, *2* and *3* in all parts of the gastrointestinal tract. *Hif-p4h-2* had significantly higher expression levels compared to *Hif-p4h-1* and *3* in colon and caecum epithelium. To test the roles each HIF-P4H isoform plays in the gut epithelium, we measured the gene expression of classical HIF target genes in *Hif-p4h-1^−/−^*, *Hif-p4h-2* hypomorph and *Hif-p4h-3^−/−^* mice. Only *Hif-p4h-2* hypomorphism led to an upregulation of HIF target genes, confirming a predominant role of HIF-P4H-2. However, the abundance of *Hif-p4h-1* and *3* expression in the gastrointestinal epithelium implies that these isoforms may have specific functions as well. Thus, the development of selective inhibitors might be useful for diverging therapeutic needs.

## 1. Introduction

The adaptation of a cell to hypoxia is regulated by hypoxia-inducible factor (HIF), which consists of an oxygen-dependent α-subunit (HIF1α, HIF2α, HIF3α) and a constitutively expressed β-subunit [1]. The HIF prolyl 4-hydroxylases (HIF-P4Hs 1, 2 and 3, also known as PHDs 1, 2 and 3 and EglNs 2, 1 and 3, respectively) control the accumulation of HIF by oxygen-dependent hydroxylation of at least one of two critical proline residues in the α-subunit [2,3]. This hydroxylation targets the HIFα subunit for rapid proteasomal degradation under normoxic conditions. In hypoxia or in the presence of chemical HIF-P4H inhibitors (P4HI), the hydroxylation is inhibited and HIFα escapes degradation, translocates into the nucleus, forms a functional dimer with HIFβ and modulates the expression of more than 300 genes involved in cellular adaptation to hypoxia [2,4]. The transcriptional activity of HIF is additionally regulated by the asparagine hydroxylase factor inhibiting HIF1α (FIH1) which prevents interaction of HIF with its transcriptional coactivator p300 [5].

HIF1α enhances the epithelial barrier function and a conditional HIF1α knockout exacerbates colitis in mouse models [6], indicating a protective role of HIF1 in the guts. HIF2α is considered to act in a rather proinflammatory manner but also to play an important part in regenerative processes [6,7,8]. This makes its role in inflammatory processes ambivalent, as its moderate activity might be desirable for optimal recovery but increase the inflammatory process at the same time. Similarly, in different tumour cell lines a deletion of HIF2α affected cell proliferation conversely, but in spontaneous colorectal cancer HIF2α seems to act rather in a protumorigenic manner, whereas a stabilization of HIF1α did not increase the tumour growth in the mouse models [6]. The activation of HIF via the inhibition of HIF-P4Hs may have vast therapeutic value in the treatment of pathological conditions characterized by insufficient O_2_ levels. The first P4HI to treat anaemia in patients suffering from a chronic kidney disease received permission for clinical use only recently [9,10].

HIF-P4H has three different isoforms [2,3] and an additional transmembrane P4H (P4H-TM) has also been shown to contribute to the regulation of HIF [11,12,13]. The relative abundance of the HIF-P4Hs and hence their contribution to the regulation of HIF stability is known to vary in different cell types [14]. Furthermore, distinct patterns of tissue expression have been reported for the HIF-P4Hs and P4H-TM and it has been postulated that the effects of the HIF-P4H isoforms are dependent on their tissue distribution [14,15].

In general, HIF-P4H-2 has been reported to be quite ubiquitously expressed, being the predominant HIF-P4H isoform in most tissues studied, for example in the heart, brain, liver, skeletal muscle and kidney, while it was detected only at low levels in the lung [15,16,17]. HIF-P4H-1 has been postulated to be the only isoform expressed in the testes [15,18], although another study demonstrated an abundance of all three isoforms in this tissue [19]. Highest HIF-P4H-3 expression has been detected in the heart [15] and HIF-P4H-3 seems to be particularly strongly expressed in epithelial cells of various tissues [19]. P4H-TM expression has been detected in the brain, eye, heart, lung, skeletal muscle and kidney, and it seems to play specific roles in neuronal tissues, e.g., the retinal pigment epithelium and astrocytes [20,21]. In the brain, P4H-TM was particularly abundant in regions related to emotional and social behaviour, which is in accordance with decreased anxiety and behavioural despair and increased social behaviour of *P4h-tm^−/−^* mice [20].

Regarding the gastrointestinal tract, only few and contradictory data concerning the expression and roles of the individual HIF-P4H isoforms exist, especially when focusing on the epithelial lining instead of the full intestinal wall that includes many different cell types. However, especially the gastrointestinal epithelium could give unique insights into the regulation of hypoxic signalling. Due to its localization at the border of the anaerobic lumen and its varying perfusion through the mesenterial vessels from the serosal side, it is usually confronted with large oxygen gradients that would not be tolerated by many other tissues. It has been established that the intestinal epithelium is able to adapt to these circumstances elaborately, being constantly in a state of “physiological hypoxia” [22]. Thus, the gut epithelium probably commands exceptional adaptation mechanisms to survive the hypoxic periods and understanding these mechanisms might hold valuable therapeutic potential.

In murine colon epithelium, a predominant expression of *Hif-p4h-3* mRNA, a nearly similar level of *Hif-p4h-2* mRNA and a far lower expression level of *Hif-p4h-1* mRNA has been reported based on RT-PCR analyses [23]. This implicates a predominant role of HIF-P4H-3 and/or 2 in the colon epithelium. In contrast to HIF-P4H-1, the expression of the latter two isoforms is induced by HIF itself [14,24]—thus, their seemingly predominant expression might be associated with the physiological hypoxia in the gastrointestinal epithelium. However, several authors have reported a crucial role of HIF-P4H-1 in inflammatory bowel disease (IBD) [25,26,27], and a therapeutic use of P4HI in IBD has been suggested [18]. Thus far, this could be associated with a less inflammatory phenotype of the immune cells [28], but there is no clear evidence whether epithelial HIF-P4H-1 plays a role as well. With respect to the P4HIs being developed at the moment, an isoform-specific inhibition may be an important next step for the treatment of pathologic conditions like IBD [29]. However, a profound understanding of the expression profile and role of each isoform in the target tissue is a prerequisite for the successful implementation of these drugs.

Therefore, we aimed to analyse the absolute abundance of the HIF-P4H isoform mRNAs in the murine gastrointestinal epithelium. In this study, we investigated the expression levels of the *Hif-p4h* isoforms *1*, *2*, *3* and *P4h-tm* as well as *Fih1*, *Hif1a* and *Hif2a* in the murine jejunum, caecum and colon epithelium applying droplet digital PCR (ddPCR). Furthermore, we characterized the relative importance of HIF-P4H-1, 2 and 3 in the regulation of selected HIF target genes using gastrointestinal samples obtained from the respective gene-modified mouse lines.

## 2. Results

### 2.1. Expression Profiles of the HIF Pathway Components in Murine Jejunum, Caecum and Colon Epithelium

To measure the absolute mRNA expression levels of the main HIF pathway components, we conducted a ddPCR analysis for *Hif1a*, *Hif2a*, *Hif-p4h-1*, *Hif-p4h-2*, *Hif-p4h-3*, *Fih1* and *P4h-tm*. Additionally, the expression levels of sodium-coupled glucose transporter 1 (*Sglt1*) and vimentin (*Vim*) were measured as markers for epithelial cells and fibroblasts, respectively, in the jejunal samples to ascertain the purity of the epithelial samples. While the expression of *Sglt1* was markedly higher than that of any other gene measured (13,256 ± 2062 copies/fg RNA input), *Vim* expression was detected at a very low level (16 ± 2 copies/fg RNA input) in all samples, indicating only a very low percentage of connective tissue contamination.

**Figure 1:**
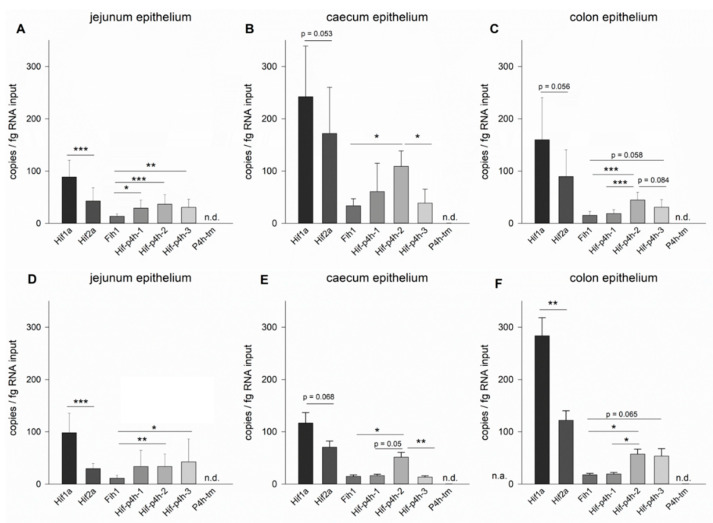
Expression of HIF pathway components in the epithelium of the murine gastrointestinal tract. mRNA expression levels *of Hif1a*, *Hif2a*, *Fih1*, *Hif-p4h* isoforms *1*, *2*, *3* and *P4h-tm* at 8 (**A**–**C**) and 16 (**D**–**F**) weeks of age in wild-type mouse jejunum (**A**,**D**), caecum (**B**,**E**) and colon (**C**,**F**) epithelium assessed by ddPCR; *n* = 14 for (**A**,**D**), and 8 for (**B**,**C**,**E**,**F**); data are presented as means ± SD, One-Way RM ANOVA and subsequent Tukey test, asterisks indicate significant differences between the genes (*Hif1a* vs. *Hif2a* and *Hif-p4h-1* vs. *2* vs. *3* vs. *Fih1*), * *p* < 0.05, ** *p* < 0.01, *** *p* < 0.001; n.d.: not detected.

Regarding the target HIF pathway genes, all of them were expressed in all parts of the gut, except for P4h-tm, which could not be detected in the gastrointestinal epithelium in our study and is thus not discussed further (Figure 1).

#### 2.1.1. Jejunum Epithelium

At 8 weeks of age, *Hif1a* expression was most abundant in the jejunum epithelium with 88 ± 32 copies/fg RNA input, which was significantly higher than the expression of *Hif2a* (42 ± 25 copies/fg RNA input) (Figure 1A). The *Hif-p4h* isoforms *1*, *2* and *3* were expressed in the jejunum epithelium at similar levels of 27 ± 16, 35 ± 19 and 29 ± 15 copies/fg RNA input (Figure 1A). *Fih1* mRNA had the lowest abundance and was significantly different from all Hif-p4h isoforms (Figure 1A). These expression levels recurred also in the older animals of 16 weeks of age (Figure 1D).

**Figure 2:**
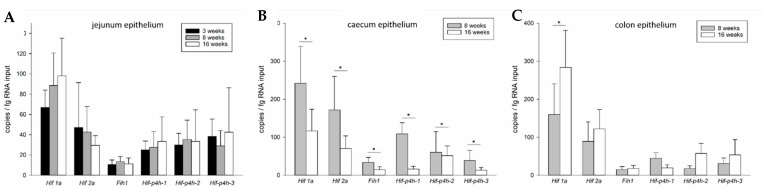
Age-dependent expression of HIF pathway components in the murine gastrointestinal epithelium. mRNA expression levels of *Hif1a*, *Hif2a*, *Fih1*, *Hif-p4h* isoforms *1*, *2*, *3* and *P4h-tm* in wild-type mouse jejunum (**A**), caecum (**B**) and colon (**C**) epithelium at different ages. Jejunum: *n* = 6 for 3 weeks and *n* = 14 for 8 and 16 weeks; colon and caecum: *n* = 8; data are presented as means ± SD, One-Way RM ANOVA and subsequent Holm-Sidak or Dunn’s test, asterisks indicate significant differences (*p* < 0.05) in the expression level of the same gene in the different age groups.

Comparing the gene expression levels in the jejunum epithelium at three different ages (Figure 2A), *Hif1a* expression seemed to increase in the older animals, but this was not statistically significant (*p* = 0.162). Corresponding to that, we observed a trend for a downregulation of *Hif2a*, but this was not significant either (*p* = 0.094). *Fih1* was expressed at steady levels in all age groups (Figure 2A). At 3 and 16 weeks of age, *Hif-p4h-3* seemed to have a slightly higher expression level than the other two isoforms, especially *Hif-p4h-1*, but no statistically significant differences between the *Hif-p4h* isoform mRNA levels were observed at any age (Figure 2A).

#### 2.1.2. Caecum Epithelium

In the caecum epithelium *Hif1a* expression reached 242 ± 97 copies/fg RNA input at 8 weeks of age and thus exceeded that of *Hif2a* (171 ± 88 copies/fg RNA input), although this difference did not quite reach statistical significance (Figure 1B). Of the *Hif-p4h* isoforms, *Hif-p4h-2* had the highest abundance (109 ± 29 copies/fg RNA input at 8 weeks of age), which was significantly higher when compared to *Hif-p4h-3* (39 ± 27 copies/fg RNA input) and *Fih1* (34 ± 13 copies/fg RNA input), while no statistical significance was reached when compared to *Hif-p4h-1* (61 ± 54 copies/fg RNA input), but the trend increased when the animals grew older (Figure 1B, E). Although the mRNA abundancies in relation to each other stayed approximately the same at 16 weeks of age (Figure 1E), the expression of all these genes decreased significantly from 8 to 16-week-old mice in the caecum epithelium (Figure 2B).

#### 2.1.3. Colon Epithelium

In the colon epithelium the expression levels of the HIF pathway components in relation to each other resembled those observed in the caecum epithelium, indicating a common pattern in the large intestines (Figure 1). In the colon epithelium of 8-week-old mice (Figure 1C), *Hif1a* mRNA was the most abundant with an expression of 160 ± 81 copies/fg RNA input, whereas the expression of *Hif2a* was 90 ± 51 copies/fg RNA input, but the difference between the *HIF* isoforms did not quite reach statistical significance at this age. *Hif-p4h-2* mRNA had the highest abundance (44 ± 15 copies/fg RNA input) among the *Hif-p4h* isoforms, which was significantly higher than that of *Hif-p4h-1* (18 ± 7 copies/fg RNA input) and *Fih1* (15 ± 8 copies/fg RNA input), while the difference between the *Hif-p4h-2* and *Hif-p4h-3* (30 ± 15 copies/fg RNA input) mRNA levels did not reach statistical significance (Figure 1C). *Fih1* mRNA level was similar to that of *Hif-p4h-1* (Figure 1C).

Comparing the two age groups (Figure 2C), a significant increase in the expression of *Hif1a* was observed from 8 to 16 weeks of age, thus enhancing the difference between *Hif1a* and *Hif2a* expression to statistical significance at 16 weeks of age (Figure 1C,F and Figure 2C). Otherwise, the mRNA expression levels of the HIF pathway components on the colon were not significantly different between the age groups (Figure 2C).

#### 2.1.4. Expression Level Differences between Small and Large Intestines

**Figure 3:**
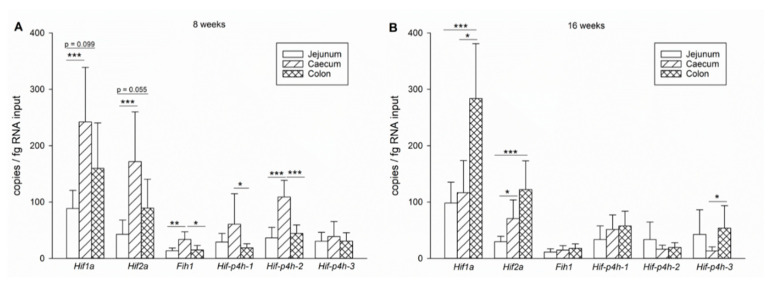
Comparative analysis of HIF pathway component expression in the small and large intestines. mRNA expression levels of *Hif1a*, *Hif2a*, *Fih1* and *Hif-p4h* isoforms *1*, *2* and *3* in jejunum, caecum and colon epithelium of 8 (**A**) and 16 (**B**) -week-old wild-type mice. *n* = 14 for jejunum and 8 for caecum and colon epithelium; data are presented as means ± SD, One-Way RM ANOVA and subsequent Dunn’s test, asterisks indicate significant differences between locations, * *p* < 0.05, ** *p* < 0.01, *** *p* < 0.001.

At 8 weeks of age, expression of all the genes except *Hif-p4h-1* and *3* was significantly higher in the caecum epithelium when compared to the jejunum epithelium (Figure 3A). Regarding the mRNA abundancies of *Hif1a* and *Hif2a*, the colon epithelium seems to be intermediate between the jejunum and caecum epithelium, while the expression of the *Hif-p4h* isoforms and *Fih1* is rather similar in both the jejunum and colon epithelium (Figure 3A). However, as noted above, at 16 weeks of age the expression levels of the HIF pathway components in the caecum epithelium were reduced, while *Hif1a* expression in the colon epithelium was increased (Figure 2B,C), leading to a significantly higher expression level of *Hif1a* in the colon than in the caecum and jejunum epithelium (Figure 3B). Combining the overall impression of the HIF pathway gene expression in the caecum and colon epithelium as the large intestinal expression versus the jejunum epithelium as the small intestine expression, it becomes evident that expression of the HIF pathway components increases from the proximal towards the distal end of the gastrointestinal tract, although age-dependent changes occur that will be discussed below.

#### 2.2. HIF-P4H-2 Plays a Major Role in the Regulation of Hypoxic Signalling in Murine Jejunum Epithelium

As none of the *Hif-p4h* isoforms struck out as a consistently dominant one in the gut epithelium at different locations and time points analysed, this brought up the question whether one isoform is more important than the others in regulating HIF stability and hence expression of HIF target genes or if all three HIF-P4H isoforms work redundantly in the gut epithelium. To assess their individual roles, we isolated jejunum and colon epithelium samples from HIF-P4H-1 and 3 knockout (*Hif-p4h-1^−/−^* and *Hif-p4h-3^−/−^*) and HIF-P4H-2 hypomorph (*Hif-p4h-2^gt/gt^*) mice and their corresponding wild-type littermates to analyse the expression of classical HIF target genes (Table 1) by RT-qPCR.

Varying levels of *Hif-p4h-2* mRNA expression ranging from 8%-85% have been detected in different tissues of the *Hif-p4h-2^gt/gt^* mice [30]. As the knockdown efficiency in the *Hif-p4h-2^gt/gt^* intestinal epithelium has not been analysed before, we first determined it using RT-qPCR, as described before [30]. The *Hif-p4h-2* expression levels were found to be 20 ± 8% in jejunum and 16 ± 8% in colon epithelium relative to the expression in wild-type mice, confirming efficient silencing of *Hif-p4h-2*.

**Figure 4:**
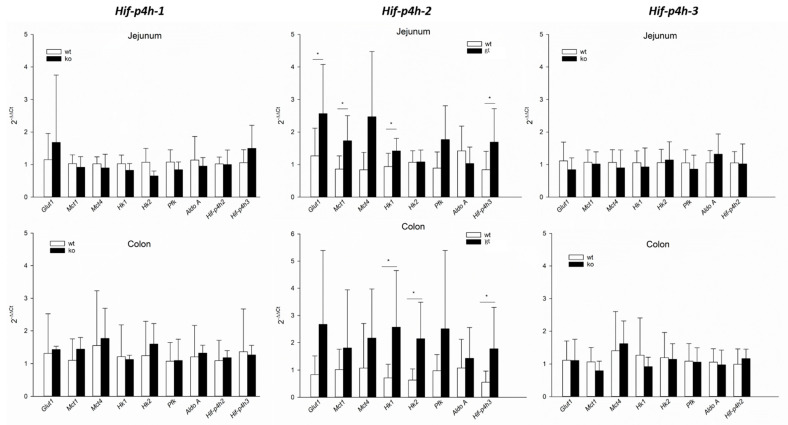
Analysis of HIF target gene expression in the gastrointestinal epithelium of *Hif-p4h* gene-modified mice. mRNA expression levels of selected HIF target genes were analysed by RT-qPCR in the jejunum (upper panel) and colon (lower panel) epithelium of *Hif-p4h-1^−/−^*, *Hif-p4h-2^gt/gt^* and *Hif-p4h-3^−/−^* mice (black bars) relative to their wild-type littermates (white bars). Data are presented as means ± SD, Student’s t-test, asterisks indicate a significant difference when compared to the wild type, * *p* < 0.05. *n* = 5 *Hif-p4h-1^+/+^* and 9 *Hif-p4h-1^−/−^* jejunum, 2 *Hif-p4h-1^+/+^* and 5 *Hif-p4h-1^−/−^* colon, 8 *Hif-p4h-2^+/+^* and 9 *Hif-p4h-2^gt/gt^* jejunum, 7 *Hif-p4h-2^+/+^* and *Hif-p4h-2^gt/gt^* colon, and 8 *Hif-p4h-3^+/+^* and *Hif-p4h-3^−/−^* jejunum and colon.

We observed that *Hif-p4h-2* hypomorphism resulted in significant induction of the mRNA expression of the HIF target genes *Glut1*, *Mct1*, *Hk1* and *Hif-p4h-3* in the jejunum epithelium and *Hk1*, *Hk2* and *Hif-p4h-3* in the colon epithelium (Figure 4). In contrast to that, the knockout of either *Hif-p4h-1* or *3* had no effect on the mRNA expression of any of the HIF target genes analysed (Figure 4), indicating a predominant role for HIF-P4H-2 in the regulation of HIF signalling in jejunum and colon epithelium.

## 3. Discussion

The gastrointestinal epithelium generally exists at very low pO_2_. This “physiological hypoxia” arises from its location next to the anaerobic gut lumen and additionally countercurrent blood flow dynamics in the intestinal villi [31,32]. Furthermore, the intestinal perfusion depends on systemic circumstances and varies largely between the postprandial and interdigestive phases. This implies that it might also have a specialised way of regulating the adaptation mechanisms to hypoxia. Thus far, only a few studies have investigated the expression profiles of the main HIF pathway components in the gastrointestinal epithelium, and most assessed their expression in whole wall homogenate, i.e., including muscles, connective tissue, and hence resulting in only small amounts of epithelial cells, which does not enable specific conclusions of the gastrointestinal epithelium. Furthermore, to our knowledge, the caecum epithelium has never been studied in this regard. With respect to the imminent role the gastrointestinal epithelium plays in keeping up the intestinal barrier and at the same time absorbing nutrients, profound knowledge of the signalling pathways supporting its integrity is crucial.

We applied the ddPCR method to assess not only relative, but also absolute expression levels of the *Hif-p4h* isoforms *1*, *2*, *3* and *P4h-tm* as well as *Fih1*, *Hif1a* and *Hif2a* in the murine jejunum, caecum and colon epithelium. We found a trend to increasing levels of nearly all HIF pathway genes from the small towards the large intestinal epithelium that seem to coincide with the oxygen levels reported for the respective locations. The measurement of pO_2_ in the gastrointestinal tract is complicated by the variations in perfusion described above and by its inaccessibility for *in vivo* measurements. Several studies that attempted to define average oxygen pressures for diverse locations along the gastrointestinal tract resulted in considerably differing values, probably due to different measurement techniques, locations, and time points. It is generally agreed that the pO_2_ decreases radially from the basolateral cell pole to the villi top in intestinal epithelial cells. Thus, measuring at the exact same position is nearly impossible, although pivotal when different parts of the gut shall be compared, which most studies do not even attempt. Concentrations (luminal) of 32 mmHg in the duodenum, ≤11 mmHg in the distal small intestine and 3 mmHg in the murine colon within the same animals have been reported [6,33], and it seems plausible that there is a general decrease in pO_2_ in the oral to aboral direction. Thus, the higher expression of *Hif1a*, *Hif2a*, *Fih1* and *Hif-p4h-1* and *2* in the large intestinal epithelium compared to the jejunum epithelium may mirror the differences in oxygen pressure and thus the need for regulative capacities. An inverse correlation of pO_2_ and the expression of the *Hif-p4hs* has been reported before, implying that cells adapt to their specific “normoxic” oxygen levels [34], i.e., physiological hypoxia, and might reflect a preconditioning to low pO_2_. This notion is additionally supported by the high amount of (facultative) anaerobic bacteria in the large intestine, which not only further reduces luminal oxygen levels but also produces substantial amounts of short chain fatty acids [35]. Short chain fatty acids, especially butyrate, have been shown to interfere with hypoxic signalling [6,36], thereby possibly enhancing the epithelial resistance to hypoxia. Hence, the differences in the HIF pathway gene expression between the different parts of the gut might reflect their diverging exposure to these fermentation products.

Another interesting finding of our study is the increase in *Hif1a* expression in colon epithelium between 8- and 16-week-old mice, a period that might be considered as the transition phase from adolescent to adult animals. In light of approximately 25% of IBD cases in humans developing during childhood or early adolescence with a primary location in the colon and an inflammatory background [37] and the increasing evidence for a role of HIF1α in the pathophysiology of IBD [25] this might be one more piece in the puzzle of IBD pathophysiology. The age-dependent expression of HIF1α should be investigated further in future studies with animals older than one year.

For the most part, the gene expression levels observed in our study confirm the general opinion that HIF-P4H-2 is the primary isoform expressed and controlling HIF stability in most tissues. Our finding that *P4h-tm* is not expressed in the gastrointestinal epithelium provides further support to the notion that this enzyme has a rather restricted expression pattern with the most abundant levels in the neuronal tissues [2,11,21,38].

Especially in the colon and caecum epithelium, a predominant expression of *Hif-p4h-2* over the other two isoforms was found, whereas in the jejunum epithelium all three isoforms were expressed at similar levels and in the 16-week-old mice there was even a trend for *Hif-p4h-3* taking the lead in jejunum and colon epithelium. The simultaneous expression of all three *Hif-p4h* isoforms in jejunum epithelium might be interpreted as a sign of functionally redundant proteins ensuring stabilization of HIF only under severe hypoxia in a tissue that undergoes hypoxia so regularly. It has been proposed that HIF-P4H isoforms have nonredundant, but still overlapping roles in the regulation of HIF depending on their relative tissue and cell type abundance [7,14]. This is in accordance with the finding that all three HIF-P4H isoforms play a role in the regulation of HIF1α [14] and that inactivation of more than one isoform was necessary to stabilize HIF1α and HIF2α in murine intestinal epithelia [7]. Contrary to that, *Hif-p4h-2* hypomorphism alone led to an increased expression of HIF target genes in our study, whereas the knockout of *Hif-p4h-1* or *3* had no effect, indicating that *Hif-p4h-2* is the primary isoform regulating HIF in the gastrointestinal epithelium. While this could be explained by the slightly predominant expression of *Hif-p4h-2* in the colon epithelium, in the jejunum epithelium all three isoforms showed similar abundancies and still only the *Hif-p4h-2* hypomorphism affected the expression of HIF target genes.

HIF-P4H-1 has been reported to be increasingly expressed in the inflamed gut epithelium [38] and there has been mounting evidence that HIF-P4H-1 plays an important role in inflammatory processes in the gut epithelium, e.g., in Crohn’s disease or ulcerative colitis [25,26,39,40] and that its inhibition could be beneficial in these conditions [18,29], while a knockout of *Hif-p4h-2* or *3* did not affect inflammatory lesions in the guts [26]. A conditional knockout of *Hif-p4h-1* did not lead to stabilization of HIF1α in general, but rather in tissues where *Hif-p4h-1* is the predominantly expressed isoform [14] and it was speculated that HIF-P4H-1 may not be involved in oxygen-sensing and the regulation of HIF1α [41]. This is supported by our findings presented here. In contrast, HIF-P4H-1 has been reported to activate nuclear factor κB (NFκB), a mediator of both inflammation and apoptosis, and thus the anti-inflammatory effect of HIF-P4H-1 inhibition could be attributed to its regulation of NFκB rather than HIF1α [42]. A direct regulation of NFκB by HIF-P4H-1 is cast into doubt, however, by the recent finding that none of the so far postulated additional hydroxylation targets of the HIF-P4Hs, including inhibitor of κB (IκB), could be confirmed to be hydroxylated by any of the HIF-P4Hs experimentally under conditions where HIFα is efficiently hydroxylated [43]. Nevertheless, the role HIF-P4H-1 seems to play in inflammatory processes is of huge interest with respect to the high prevalence of IBD in modern society. The genuine expression of *Hif-p4h-1* in the gut epithelium, while missing any effect on the HIF pathway as observed in our study supports the notion that it probably plays a HIF-independent regulative role in the intestinal epithelium.

In summary, we evaluated for the first time the absolute expression levels of *Hif-p4h-1*, *2*, *3* and *P4h-tm* as well as *Fih1*, *Hif1a* and *Hif2a* in the epithelium of all compartments of the murine gastrointestinal tract. Our findings support a central role of HIF-P4H-2 in the regulation of HIF1α in the gastrointestinal epithelium. However, as existing evidence suggests that HIF-P4H-1 and 3 are nonetheless crucial factors for epithelial integrity involving, for example, NFκB and HIF2α, more studies are needed to understand their individual roles in gastrointestinal homeostasis and in various disease conditions affecting this tissue. Such information would be extremely valuable to assess the possible necessity of development of selective inhibitors for clinical use.

## 4. Materials and Methods

### 4.1. Animal Experiments

C57BL/6N mice of 3, 8 and 16 weeks of age were used for mRNA expression analysis in the jejunum, and mice of 8 and 16 weeks of age for the colon and caecum epithelium. Only the jejunum epithelium could be sampled from the 3-week-old mice due to the small size of their intestines. The mice were anesthetized and sacrificed with a CO_2_ flow at 20% of the cage volume/minute. Death was ascertained by cervical dislocation before opening the abdominal cavity and excising the intestines. The intestines were cut open longitudinally and epithelium of the jejunum, caecum and colon was isolated by scraping it off the muscular layers after washing in PBS twice. The samples were snap frozen in liquid nitrogen and stored at −80 °C.

The intestinal epithelium was sampled as described above also from HIF-P4H-1 and 3 knockout (*Hif-p4h-1^−/−^*, *Hif-p4h-3^−/−^*) and HIF-P4H-2 hypomorph (*Hif-p4h-2^gt/gt^*) mice [8,30]. Wild-type littermates were used as controls.

The mice were fed a standard-chow diet and water *ad libitum*. Mice of both sexes were used in approximately equal amounts in each experimental group. The mice were derived from the breeding barrier and experiments were carried out in the conventional unit of the Oulu Laboratory Animal Centre. In biannual microbiological monitoring of the conventional unit for the primary mouse pathogens listed in the Federation of European Laboratory Animal Science Associations (FELASA) recommendations [44], all results were negative apart from mouse norovirus 18 months before and during the study. The Animal Experiment Board of Finland, following the regulations of the EU Directive 86/609/EEC, the European Convention ETS123, and the national legislation of Finland, approved the animal experiments in this study (permission valid from 13.12.2017-12.12.2021). Recommendations concerning laboratory animal experiments and handling given by the FELASA, and the Finnish and EU legislations were followed.

### 4.2. mRNA Extraction and PCR Analysis

Total mRNA was extracted from 30 mg of frozen tissue using the E.Z.N.A.^®^ Total RNA Kit I (Omega BioTek, Norcross, GA, USA). RNA quality and quantity were determined using a Nanodrop 2000 (Thermo Fisher Scientific, Waltham, MA, USA) and 1 µg of high-quality RNA was used for synthesis of cDNA using the iScript cDNA Synthesis Kit (Bio-Rad, Hercules, CA, USA) at a total volume of 20 µL. The efficiency of cDNA synthesis at equal levels in all samples was verified by qPCR using the reference genes hypoxanthine-guanine phosphoribosyltransferase 1 (*Hprt1*), peptidylprolyl isomerase A (*Ppia*) and β-actin (*Actb*) (see Table 1). The cDNA was used in a dilution of 1:20.

For ddPCR analysis, the ddPCRTM Supermix for Probes, droplet generation oil, gaskets and all other equipment were purchased from Bio-Rad. TaqMan™ Gene Expression Assays for mouse *Hif1a*, *Hif2a*, *Fih1*, *Hif-p4h-1*, *Hif-p4h-2*, *Hif-p4h-3* and *P4h-tm* as well as sodium-coupled glucose transporter 1 (*Sglt1*) and vimentin (*Vim*) as a control for purity of jejunum epithelium and contamination of the samples with connective tissue, respectively, were commercially available from ThermoFisher Scientific (Table 2). The QX200^TM^ Droplet Digital^TM^ PCR system (Bio-Rad) was used for generation and analysis of the droplets, while PCR was conducted in a C1000 Touch Thermal Cycler (Bio-Rad). TaqMan expression assay performances were tested using cDNA dilution series and matching changes in droplet numbers were observed. The results are displayed as copies/fg RNA input.

**Table 1:** Primers for qPCR.

Gene Name	5′–3′ Sequence	3′–5′ Sequence
*Aldoa*	AGTCCACCGGAAGCATTGC	CAGCCCCTGGGTAGTTGTC
*Actb*	AGAGGGAAATCGTGCGTGAC	CAATAGTGATGACCTGGCCGT
*Glut1*	TCAAACATGGAACCACCGCTA	AAGAGGCCGACAGAGAAGGAA
*Hif-p4h-2*	GCGTCCCAGTCTTTATTTAGATA	CTGGGCAACTACAGGATAAAC
*Hif-p4h-3*	GCTGGGCAAATACTATGTCAAG	CTTATTCAGGTAGTAGATACAGGTGATACA
*Hk1*	GAGTCTGAGGTCTACGACACC	CCCACGGGTAATTTCTTGTCC
*Hk2*	GTGTGCTCCGAGTAAGGGTG	CAGGCATTCGGCAATGTGG
*Hprt1*	TCAGTCAACGGGGGACATAAA	GGGGCTGTACTGCTTAACCAG
*Mct1*	TGTTAGTCGGAGCCTTCATTTC	CACTGGTCGTTGCACTGAATA
*Mct4*	TCACGGGTTTCTCCTACGC	GCCAAAGCGGTTCACACAC
*Pfk*	TGCAGCCTACAATCTGCTCC	GTCAAGTGTGCGTAGTTCTGA
*Ppia*	GAGCTGTTTGCAGACAAAGTTC	CCCTGGCACATGAATCCTGG

Quantitative real-time PCR (RT-qPCR) analysis was performed using the iTaq^TM^ Universal SYBR^®^ Green Supermix (Bio-Rad) and a CFX96 Touch Cycler (Bio-Rad) with the following protocol: 3 min 95 °C, 40 cycles of 15 s 95 °C and 45 s 60 °C, 5 min 95 °C. For each sample, two technical replicates were measured. *Hprt1*, *Ppia* and *Actb* were used as reference genes. In the *Hif-p4h-2* hypomorph mice, *Hprt1* proved not to be stably expressed, therefore in the *Hif-p4h-2^gt/gt^* and *Hif-p4-h2^wt/wt^* mice only *Ppia* and *Actb* were used as reference genes. The geometric mean of the C_t_ values of all reference genes was used for normalization. The hypoxia response target genes analysed were glucose transporter 1 (*Glut1*), monocarboxylate transporter 1 and 4 (*Mct1*, *Mct4*), aldolase A (*Aldoa*), hexokinase 1 and 2 (*Hk1*, *Hk2*), phosphofructokinase (*Pfk*) and *Hif-p4h-2* and *3* (Table 1).

### 4.3. Statistics

Unless stated otherwise, the results are described as arithmetic means ± standard deviation (SD). The significance is expressed as the probability of error (*p*). *n* values (see figure legends) represent the number of biological replicates for each sample. The data were pooled for statistical analysis. The differences between the mean values of two or more groups were tested using Student’s t-test or Repeated Measures One Way analysis of variance (ANOVA) with a subsequent Tukey, Holm-Sidak or Dunn’s test as appropriate (Sigma Plot 13.0, Systat Software Inc., Frankfurt am Main, Germany). The differences were considered statistically significant when *p* < 0.05.

## Figures and Tables

**Table 2:** Probes for ddPCR.

Gene Name	Order No.
*Hif1a*	Mm00468869_m1
*Hif2a*	Mm01236112_m1
*Fih1*	Mm01198376_m1
*Hif-p4h-1*	Mm00519067_m1
*Hif-p4h-2*	Mm00459770_m1
*Hif-p4h-3*	Mm00472200_m1
*Hif-p4h-tm*	Mm00512331_m1
*Sglt1*	Mm00451203_m1
*Vim*	Mm01333430_m1

## Data Availability

Data are available upon request from the authors.
